# Estimation of radiation dose from ingested tritium in humans by administration of deuterium-labelled compounds and food

**DOI:** 10.1038/s41598-021-82460-5

**Published:** 2021-02-02

**Authors:** Tsuyoshi Masuda, Toshitada Yoshioka

**Affiliations:** 1 Institute for Environmental Sciences, Aomori, Japan; 2 Hirosaki Gakuin University, Aomori, Japan

**Keywords:** Public health, Nuclear energy

## Abstract

Radiation doses from organically bound tritium (OBT) in foods have been a major concern near nuclear facilities. The current dose coefficient for OBT is calculated using a standard model from the International Commission on Radiological Protection, in which some biokinetic values are not based on human metabolic data. Here, the biokinetics of ingested OBT, and radiation doses from them, were estimated by administering labelled compounds and foods to volunteers, using a deuterium (D) tracer as a substitute for tritium. After the administration of D-labelled glucose, alanine, palmitic acid, or soybean, the D/H ratios in urine were measured for up to 119 days, and the biokinetic parameter values were determined for OBT metabolism. The slow degradation rates of OBT could not be obtained, in many volunteers administered glucose and alanine. The estimated committed effective dose for 1 Bq of tritium in palmitic acid varied from 3.2 × 10^–11^ to 3.5 × 10^–10^ Sv Bq^−1^ among volunteers and, for those administered soybean, it varied from 1.9 × 10^–11^ to 1.8 × 10^–10^ Sv Bq^−1^. These results suggest that OBT, present in some ingested ingredients, gives higher doses than the current dose coefficient value of 4.2 × 10^–11^ Sv Bq^−1^.

## Introduction

Tritium is released from facilities such as nuclear power plants, especially heavy water reactors and spent nuclear fuel reprocessing plants, under normal operating conditions. In 2008, the average global annual release of tritium from nuclear facilities, into atmosphere and water, was estimated to be 11.7 PBq and 16.0 PBq, respectively^1^. In the future, a significant increase is anticipated in the release of tritium from nuclear fusion reactors, such as the International Thermonuclear Experimental Reactor (ITER). Although most of the released tritium is inorganic, in the form of tritiated water (HTO) and gaseous, tritiated hydrogen (HT), part of the released tritium is incorporated into organic molecules in plants and animals, and then transferred to humans via food ingestion, as organically bound tritium (OBT)^2,3^; OBT refers in this paper to carbon-bound tritium in organic molecules formed in living systems by natural biological processes^4^. Therefore, near such nuclear facilities, the dose from ingested OBT is a public concern.

**Figure 1:**
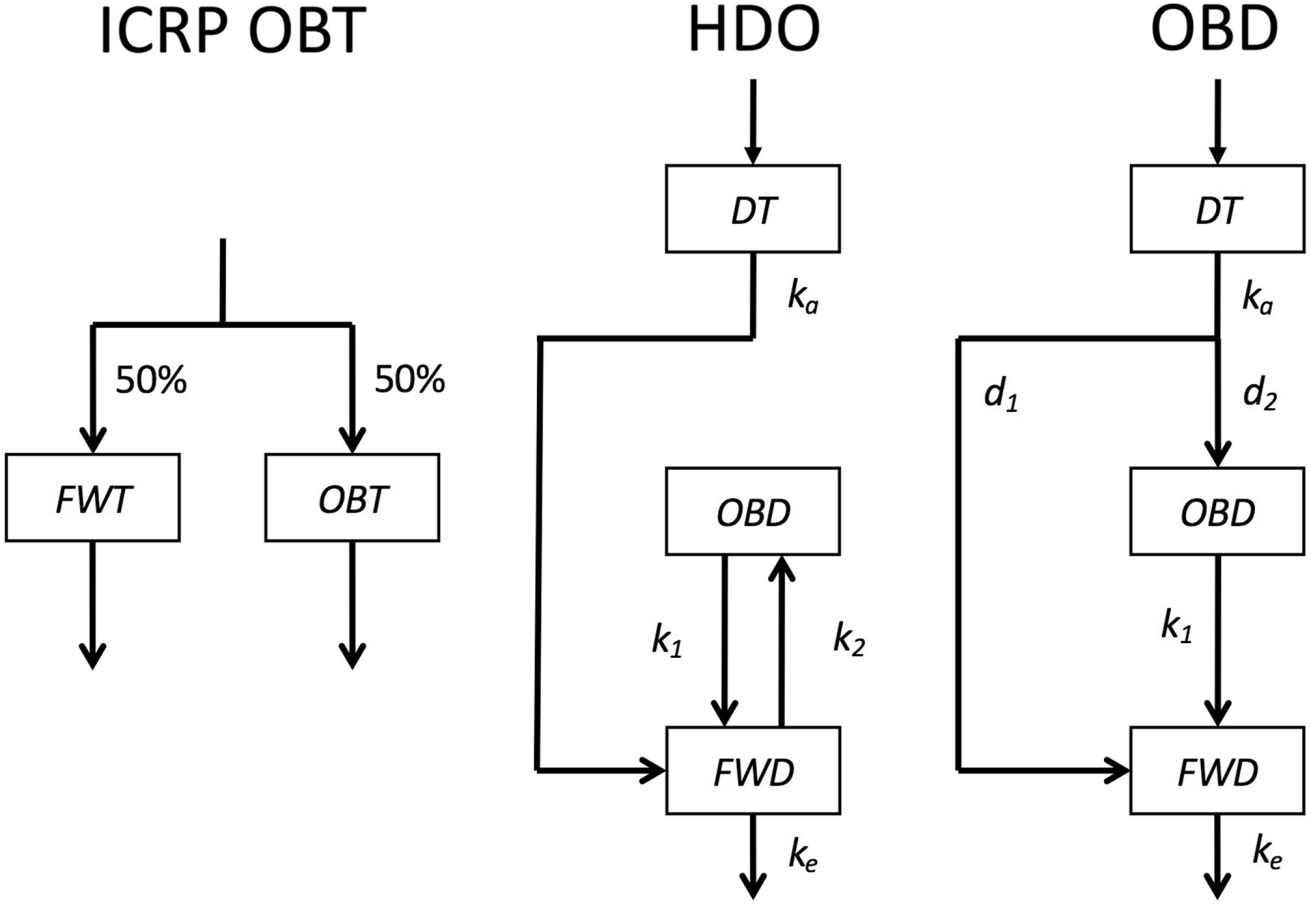
Structure of metabolic model of hydrogen isotopes. ICRP OBT is the model adopted by the International Commission on Radiological Protection (ICRP) to calculate the dose coefficient for ingested OBT^5^. HDO and OBD, the models developed to analyse the amount of D excreted through urine after administration of D-labelled compounds and food. FWT, free water tritium; OBT, organically bound tritium; DT, digestive tract; OBD, organically bound deuterium; FWD, free water deuterium; *d*_*m*_, distribution factor, *k*_*m*_, excretion rate constant (day^−1^).

In order to estimate the potential effect of ingestion of OBT, the dose coefficient is used: the ratio of the 50-year committed effective dose for 1 Bq of ingested OBT. The International Commission on Radiological Protection (ICRP) has utilised a two-compartment metabolic model, shown in Fig. 1, to calculate the dose coefficient of OBT for members of the public^5^, although it has adopted a more complex model to calculate the value for workers^4^. The ICRP OBT model consists of free water tritium (FWT) and OBT compartments, with residual half-lives of 10 and 40 days, respectively. In this model, 50% of ingested OBT is distributed to the FWT compartment by immediate metabolic degradation, while the rest of the ingested OBT is distributed to the OBT compartment^5^. Studies that provide data supporting the choice of these values (10 days, 40 days, and 50%) are important from the viewpoint of nuclear safety for members of the public. However, there has been a lack of metabolic data from humans, as described below.

Many studies have examined the half-life of FWT in humans. Butler and Leroy reported values from 310 cases, in 1965, ranging from 4 – 18 days, with a mean of 9.5 days^6^. Based on fewer cases, other studies have reported mean half-lives of 7.5, 8.5, 9.5, 9.5, 10.5, and 11.3 days^7–12^. Hill and Johnson reviewed these data and reported a mean half-life of 9.4 days, with lower and upper 90% confidence intervals of 5 and 13 days, respectively^13^. These results, obtained from humans, have supported the use of the ‘10-day’ value for half-life of FWT, as used in the existing ICRP OBT model.

However, few studies in humans have examined the biokinetics of tritium derived from ingested OBT. Many studies have reported the biokinetics of tritium in rodents, after the administration of OBT in foods such as wheat^14^, rice^15^, and soybean^15^, or in the component molecules of major nutrients, such as amino acids^16–19^, fatty acids^17^, and carbohydrates^17–19^. Hunt et al.^20^ examined the retention time of tritium in urine from volunteers after the administration of flesh from fish procured from a fisherman in Cardiff Bay (UK), which was contaminated with discharged tritium from the GE Healthcare plant. They reported the retention function as having a fast-decreasing component, with half-lives ranging from 4 to 11 days, which they considered to correspond to the FWT compartment. A second component, with a longer half-life, corresponding to the OBT compartment, was not detected due to the background fluctuation^20^. Since there have been few biokinetic studies on OBT in humans, the half-life of tritium in the OBT compartment (40 days) used in the ICRP OBT model, was calculated by the excretion rate derived from the mass balance data for carbon (16 kg of body carbon and 300 g d^−1^ of intake/excreted carbon) in a reference man^21^. This is because the tritium bound to carbon is fixed until the compound is metabolically degraded to HTO and carbon dioxide; therefore, the metabolic rate of OBT is considered to be approximately equal to that of organic carbon.

The distribution ratio of ingested OBT to the FWT compartment in the ICRP model (50%) is not based on experimental metabolic data for OBT, or organic carbon, in humans. This ratio was considered a conservative assumption, from a regulatory perspective, based on metabolic data obtained from animals such as mice^22^, rabbits^23^, rats^24^, and kangaroo rats^25^. For a more realistic estimation of dose from ingested OBT, we need experimental data from humans^26,27^.

In this paper, we report the results of experiments with administered deuterium (D)-labelled compounds as follows: D_2_O, D-labelled glucose, D-labelled alanine, D-labelled palmitic acid, and D-labelled soybean. Deuterium is a stable isotope of hydrogen. Isotopes have an almost identical rate of chemical reaction. However, this can differ significantly in cases where the molecular weights of molecules involved in the reaction are relatively small. Priest et al. examined the retention time of tritium in mice, after the administration of various tritiated compounds; in biokinetics, no difference was observed between HTO and DTO^16^. Since, in common with tritiated compounds, the molecular weights of molecules with organically bound deuterium (OBD) are higher than that of inorganic molecules, the biokinetics of OBD are considered the same as those of OBT. Therefore, deuterium labelling was used as a substitute for tritium labelled compounds and foods, for the study of metabolic rates of OBT.

## Results and discussion

### D/H ratios in urine and ^13^C/^12^C ratios in breath

**Figure 2:**
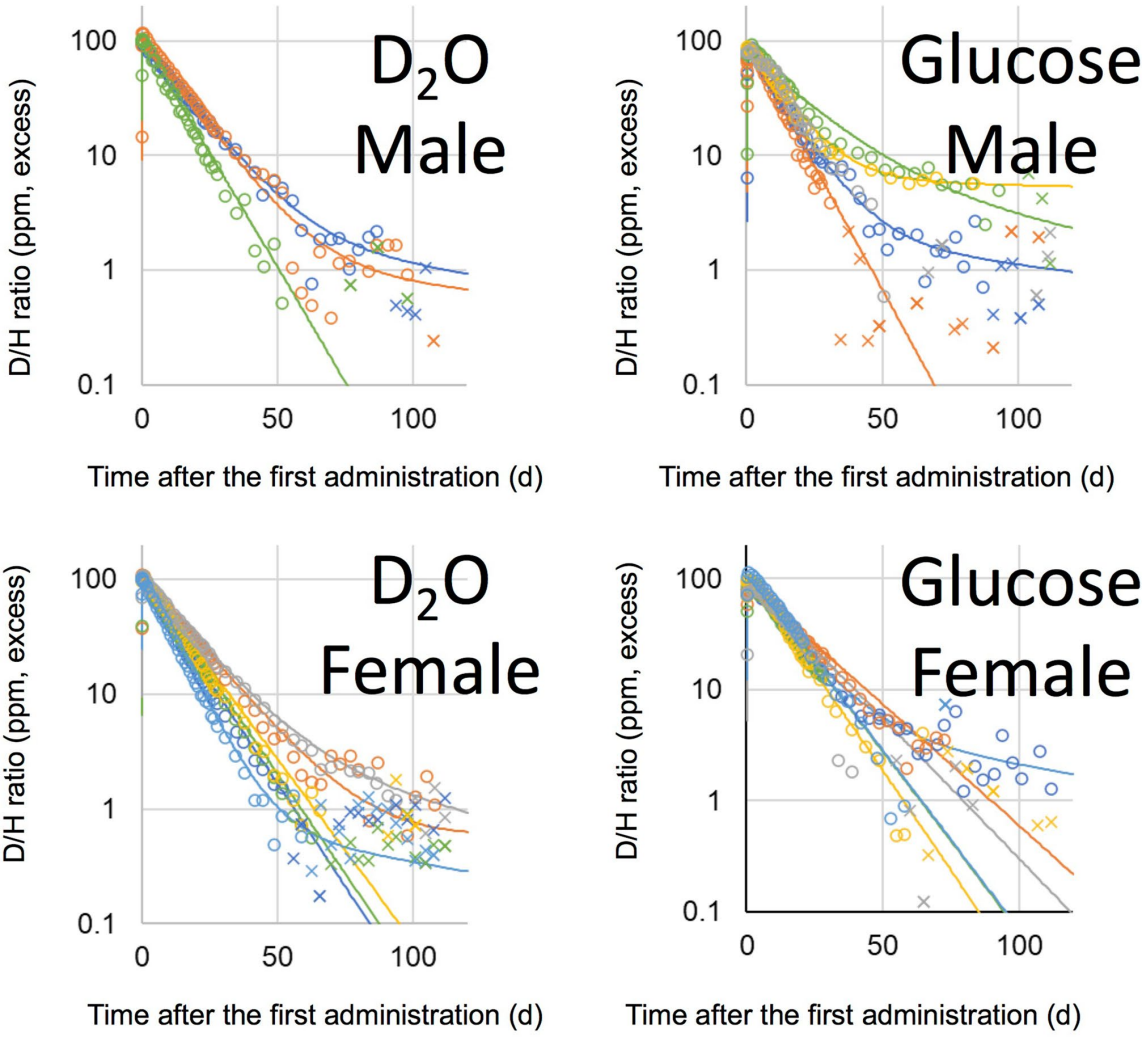
D/H ratios in urine after single oral administration of D_2_O and D-labelled glucose. The ratio was normalized to the same dosage per body weight (1 g D per 70 kg). Lines were fitted using models shown in Fig. 1. Data below the 90% upper confidence limit of the background fluctuation for each volunteer, and the subsequent data after elimination from the fit, are shown by crosses. Each colour represents a different volunteer.

**Figure 3:**
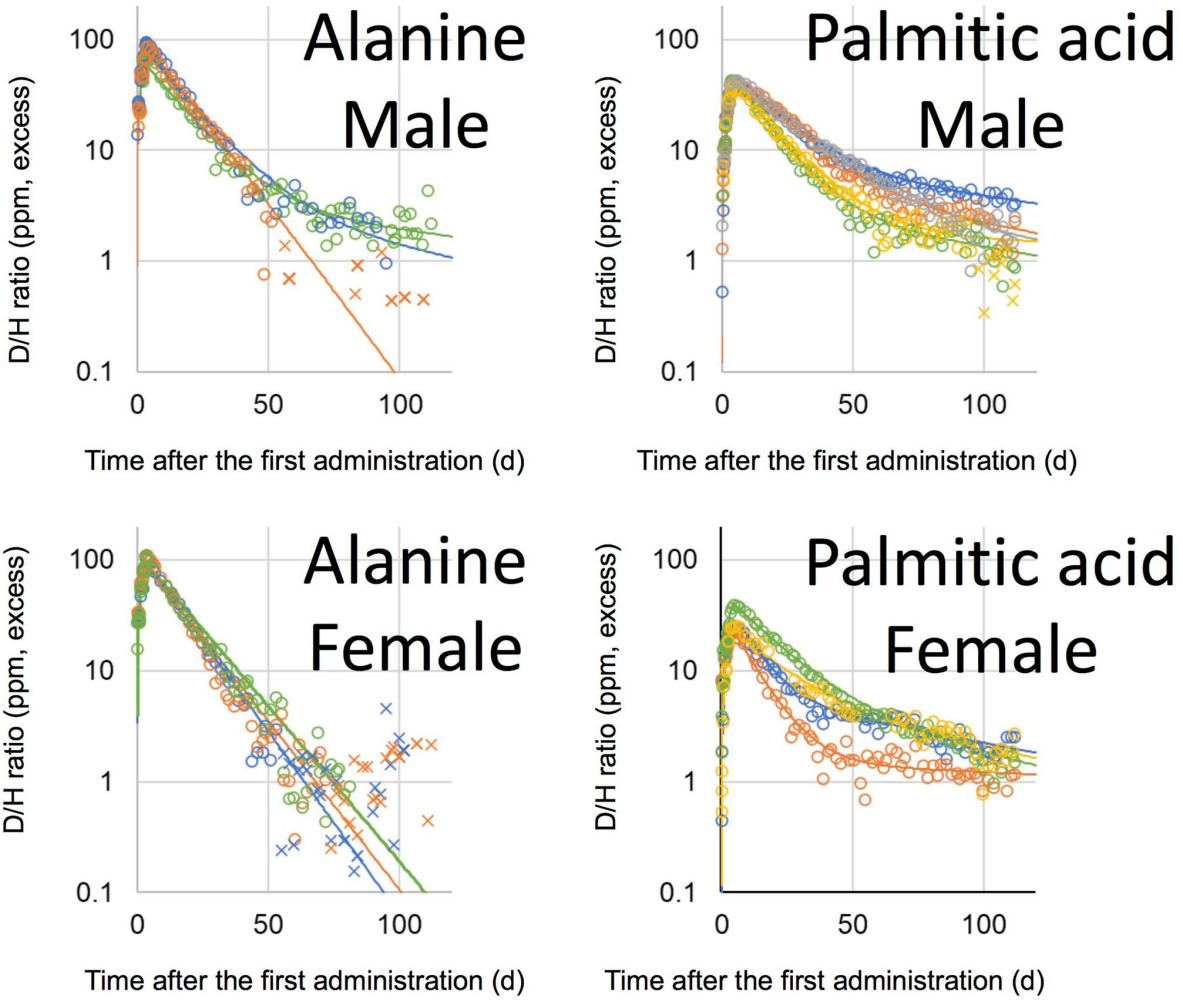
D/H ratios in urine, after oral administration of D-labelled alanine and palmitic acid, over four successive days. The ratio was normalized to the same dosage per body weight (1 g D per 70 kg). Lines were fitted using models shown in Fig. 1. Data below the 90% upper confidence limit of the background fluctuation for each volunteer, and the subsequent data after elimination from the fit, are shown by crosses. Each colour represents a different volunteer.

**Figure 4:**
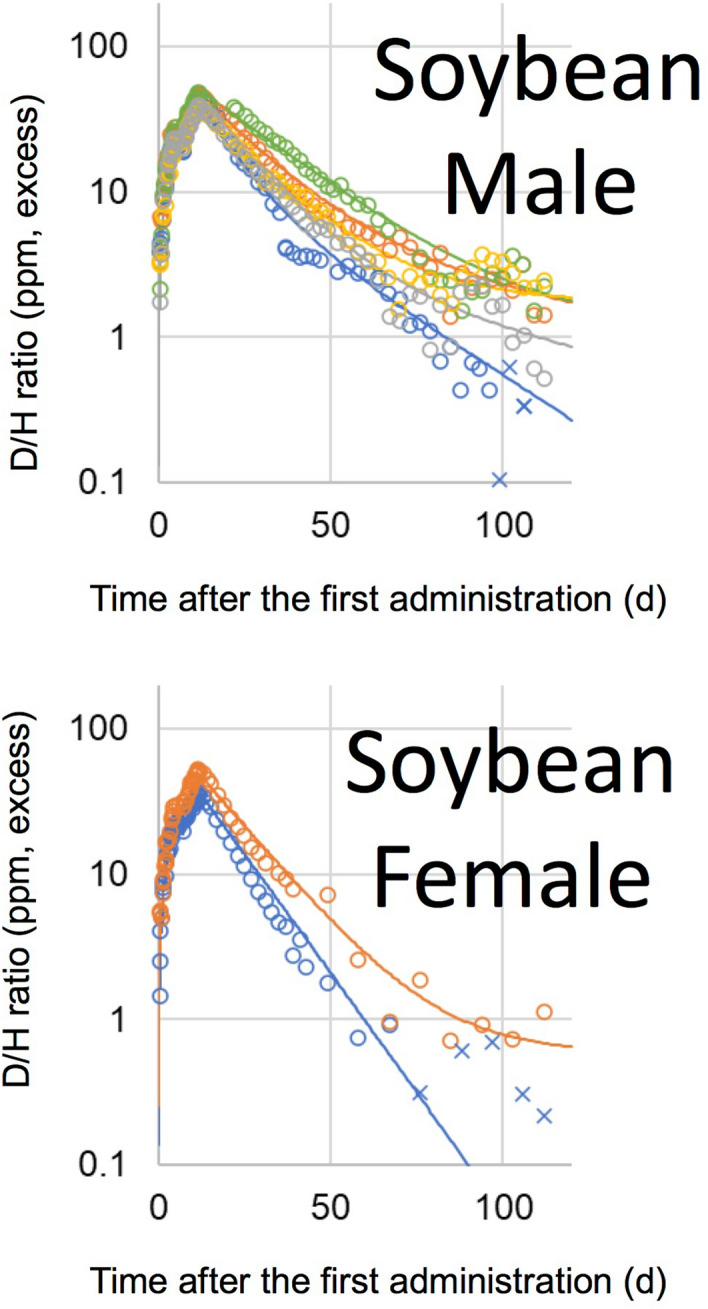
D/H ratios in urine, after oral administration of D-labelled soybean for 2 weeks, except for Saturday and Sunday. The ratio was normalized to the same dosage per body weight (1 g D per 70 kg). Lines were fitted using models shown in Fig. 1. Data below the 90% upper confidence limit of the background fluctuation for each volunteer, and the subsequent data after elimination from the fit, are shown by crosses. Each colour represents a different volunteer.

**Figure 5:**
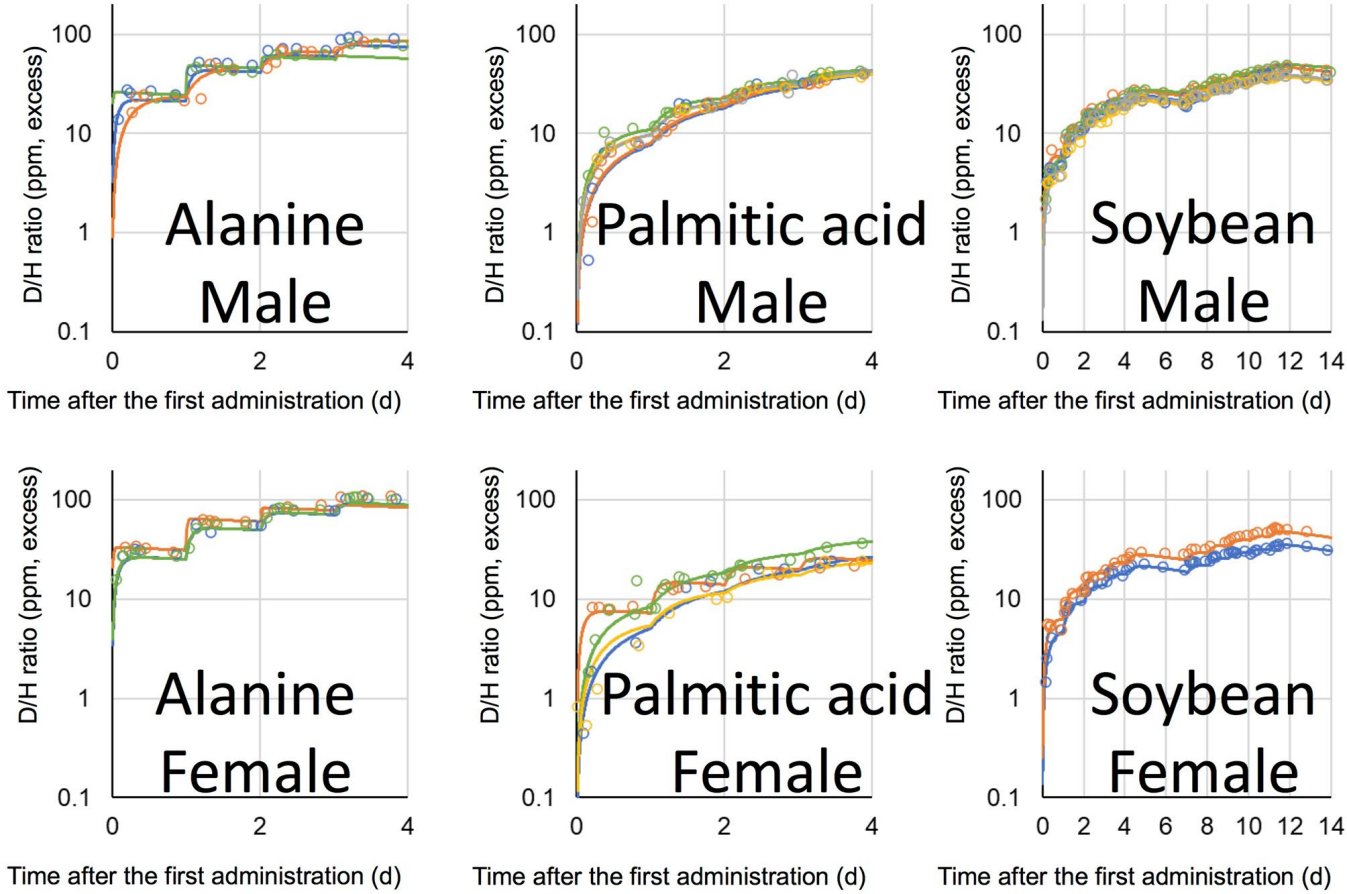
D/H ratios in urine after the oral administration of D-labelled alanine, palmitic acid, and soybean for 4, 4, and 14 days, respectively; all panels are the enlargement of the same data shown in Figs. 3 and 4. The ratio was normalized to the same dosage per body weight (1 g D per 70 kg). Lines were fitted using a model shown in Fig. 1. Each colour represents a different volunteer. D-labelled alanine and palmitic acid were administered over four successive days. D-labelled soybean was administered daily for 2 weeks, except for Saturday and Sunday.

**Figure 6:**
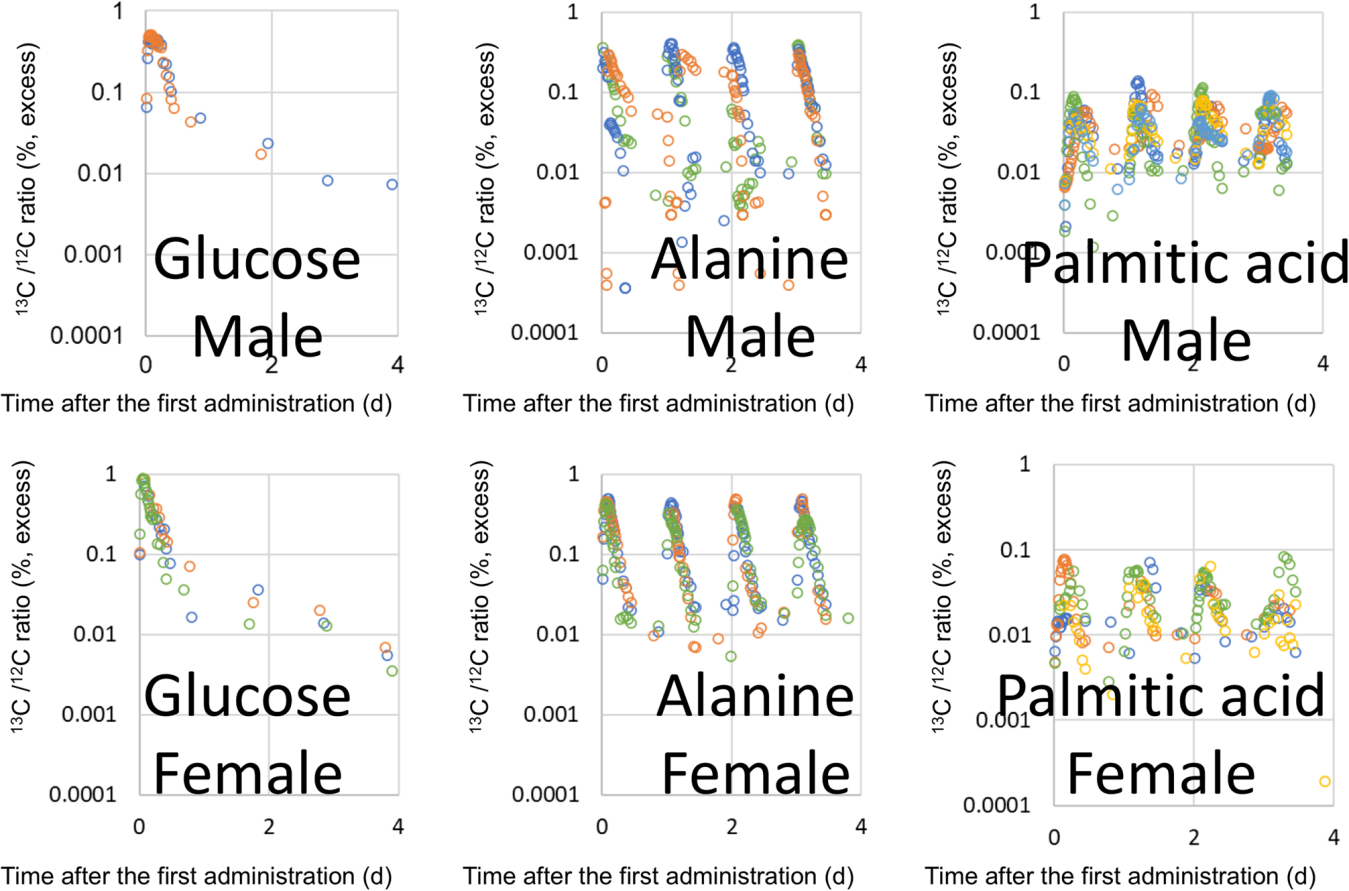
^13^C/^12^C in breath after the oral administration of ^13^C -labelled glucose, alanine, and palmitic acid. Each colour represents a different volunteer. ^13^C -labelled alanine and palmitic acid were administered over four successive days. ^13^C -labelled glucose soybean was administered once at day 0.

D_2_O and D-labelled glucose were administered once to volunteers, orally, while D-labelled alanine and D-labelled palmitic acid were administered once daily for four successive days. The D/H ratios in urine, after the administration of D_2_O and D-labelled glucose, are shown in Fig. 2, and those of D-labelled alanine and D-labelled palmitic acid are shown in Fig. 3. Boiled green soybean, which was cultivated hydroponically in 20% D_2_O solution, was administered as D-labelled soybean on weekdays, for 2 weeks, and the subsequent D/H ratios in urine are shown in Fig. 4. It is noteworthy that, in all figures showing D/H ratios in urine, the ratios are shown as increments above the background values measured before administration, and are normalised to the same dosage per body weight (1 g D per 70 kg). After the administration of D_2_O and D-labelled glucose, the D/H ratios increased and then decreased, within two exponential components, in 2 of 3 male volunteers and 2 of 6 female volunteers in the D_2_O group, and in 3 of 5 males and 1 of 5 female volunteers in the glucose group. However, the second component was not detected for the rest of the volunteers because of the rapid decrease in the ratios to below the limit (90% upper confidence limit of the background fluctuation of urine samples from each volunteer obtained before the administration) to cut off data and any subsequent data. The D/H ratios in urine, during the administration of D-labelled alanine, palmitic acid, and soybean, increased at each time point (Fig. 5). After the peak of the last administration, the ratios decreased exponentially within one or both components (Figs. 3, 4) similarly to changes observed in volunteers administered with D_2_O and D-labelled glucose. For the volunteers with D-labelled glucose, alanine, and palmitic acid, the ^13^C-labelled compounds were administered simultaneously. The excess of the ^13^C/^12^C ratios in breath, above the background ratio, were normalised to the same dosage per body weight (1 g^13^C per 70 kg), and are shown in Fig. 6. The ^13^C/^12^C ratios of alanine and palmitic acid showed steep peaks, in contrast to the gradual increase in D/H ratios for volunteers given these compounds. The half-life of FWT is significantly higher than the reported half-life of carbon dioxide measured by intravenous injection of ^14^C-bicarbonate (5 min)^28^. The difference between the D/H and ^13^C/^12^C ratios was considered to be due to the difference in the metabolisms of HDO and ^13^CO_2_, because the metabolic degradation rates of D-labelled and ^13^C-labelled compounds were considered the same until they degraded into inorganic molecules. The degradation ratio for OBT and organic carbon is assumed as the same by the ICRP^5^.

### Biokinetics of deuterium ingested as D_2_O

We developed metabolic models to analyse the data obtained from each volunteer administered D-labelled compounds or food. The structure of these models is shown in Fig. 1. The parameter values in the metabolic model for each volunteer were determined by the least squares method, and are summarised in Tables 1, 2, 3, 4 and 5.

**Table 1:** Estimated parameters of HDO model for each D_2_O administered volunteer.

Parameter	Volunteer														
	Male					Female								All	
	1	2	3	Average	S.D	1	2	3	4	5	6	Average	S.D	Average	S.D
*k*_*a*_ (d^−1^)	39	8.6	21	23	15	92	14	6.4	21	8.9	23	28	32	26	26
*k*_*e*_ (d^−1^)	0.059	0.063	0.090	0.071	0.017	0.081	0.043	0.078	0.072	0.056	0.098	0.071	0.020	0.071	0.018
*k*_1_ (d^−1^)	0.0091	0.0083	–	0.0087	0.0005	–	0.019	–	–	0.0067	0.0074	0.011	0.007	0.010	0.005
*k*_2_ (d^−1^)	0.0092	0.0065	–	0.0079	0.0020	–	0.0017*	–	–	0.016	0.010	0.013	0.007	0.010	0.0053
*T*_1*/*2_* ka* (d^−1^)	0.018	0.080	0.033	0.044	0.032	0.0075	0.051	0.11	0.033	0.078	0.030	0.051	0.037	0.049	0.034
*T*_1*/*2_* k*_*e*_ (d^−1^)	12	11	7.7	10	2	8.6	16	8.9	9.6	12	7.1	10	3	10	3
*T*_1*/*2_* k*_1_ (d^−1^)	76	83	–	80	5	–	36	–	–	104	94	78	36	79	26
*T*_1*/*2_* k*_2_ (d^−1^)	75	107	–	91	23	–	–	–	–	44	68	56	17	74	26
*R*_*r*_	0.91	1.0	0.92	0.94	0.05	0.70	0.97	0.97	0.93	1.0	0.99	0.93	0.12	0.93	0.10

S.D., standard deviation. *k*_*a*_, transfer rate from digestive tract (DT) compartment to free water deuterium (FWD) compartment (d^−1^); *k*_1_, transfer rate from organically bound deuterium (OBD) compartment to FWD compartment (d^−1^); *k*_2_, transfer rate from FWD compartment to OBD compartment (d^−1^); *k*_*e*_, excretion rate from FWD compartment (d^−1^); *T*_1*/*2_* km*, half-life of *k*_*m*_ (d); *R*_*r*_, recovery ratio to the administered deuterium. The structure of the HDO model and the parameters are shown in Fig. 1. Parameter values were determined by the least squares method except for the value accompanied with asterisk. *Presumed value of data that did not show a clear indication of the second component of exponential decrease.

**Table 2:** Estimated parameters of OBD model for each volunteer administered with D-labelled glucose.

Parameter	Volunteer																
	Male							Female								All	
	1	2	3	4	5	Average	S.D	1	2	3	4	5	6	Average	S.D	Average	S.D
*d*_1_	0.77	0.85	0.73	0.18	0.99	0.70	0.31	0.73	0.81	0.98	1.0	0.88	1.0	0.90	0.11	0.81	0.23
*d*_2_	0.23	0.0	0.27	0.81	0.0	0.26	0.33	0.27	0.0	0.0	0.0	0.0	0.0	0.045	0.110	0.14	0.25
*k*_*a*_ (d^−1^)	3.3	5.3	12	501	3.6	105	222	77	11	5.9	8.8	5.6	11	20	28	59	148
*k*_*e*_ (d^−1^)	0.085	0.097	0.050	0.085	0.078	0.079	0.017	0.073	0.049	0.072	0.081	0.057	0.072	0.067	0.012	0.073	0.015
*k*_1_ (d^−1^)	0.0070	–	0.010	0.0017*	–	0.0062	0.0047	0.0095	–	–	–	–	–	0.0095	–	0.0070	0.0041
*T*_1*/*2_* ka* (d^−1^)	0.21	0.13	0.059	0.0014	–	0.10	0.09	0.0090	0.065	0.12	0.078	0.12	0.060	0.076	0.042	0.086	0.062
*T*_1*/*2_* k*_*e*_ (d^−1^)	8.1	7.1	14	8.2	8.9	9.2	2.6	9.5	14	9.6	8.6	12	9.6	11	2	10	2
*T*_1*/*2_* k*_1_ (d^−1^)	100	–	66	–	–	83	23	73	–	–	–	–	–	73	–	80	17
*Rr*	1.0	0.85	1.0	0.99	0.99	0.96	0.07	1.0	0.81	0.98	1.0	0.88	1.0	0.95	0.08	0.95	0.07

S.D., standard deviation. *k*_*a*_, transfer rate from digestive tract (DT) compartment to free water deuterium (FWD) and organically bound deuterium (OBD) compartments (d^−1^); *k*_1_, transfer rate from OBD compartment to FWD compartment (d^−1^); *k*_*e*_, excretion rate from FWD compartment (d^−1^); *T*_1*/*2_* k*_*m*_, half-life of *k*_*m*_ (d); *R*_*r*_, recovery ratio to the administered deuterium; *d*_1_, distribution factor to FWD compartment; *d*_2_, distribution factor to OBD compartment. The structure of the OBD model and the parameters are shown in Fig. 1. Parameter values were determined by the least squares method except for the value accompanied with asterisk. *Presumed value of data that did not show a clear indication of the second component of exponential decrease.

**Table 3:** Estimated parameters of OBD model for each volunteer administered with D-labelled alanine.

Parameter	Volunteer											
	Male					Female					All	
	1	2	3	Average	S.D	1	2	3	Average	S.D	Average	S.D
*d*_1_	0.82	0.94	0.67	0.81	0.14	1.0	1.0	0.99	1.0	0.01	0.90	0.13
*d*_2_	0.18	0.0	0.33	0.17	0.16	0.0	0.0	0.0	0.0	0.0	0.085	0.14
*k*_*a*_ (d^−1^)	15	3.6	112	44	60	13	79	15	35	37	39	45
*k*_*e*_ (d^−1^)	0.065	0.071	0.068	0.068	0.003	0.074	0.068	0.063	0.069	0.006	0.068	0.004
*k*_1_ (d^−1^)	0.011	–	0.0066	0.0088	0.0031	–	–	–	–	–	0.0088	0.0031
*T*_1*/*2_* ka* (d^−1^)	0.047	0.19	0.0062	0.082	0.098	0.055	0.0088	0.046	0.036	0.024	0.059	0.068
*T*_1*/*2_* k*_*e*_ (d^−1^)	11	9.8	10	10	0	9.3	10	11	10	1	10	1
*T*_1*/*2_* k*_1_ (d^−1^)	63	–	105	84	29	–	–	–	–	–	84	29
*Rr*	1.0	0.94	1.0	0.98	0.03	1.0	1.0	0.99	1.0	0.01	0.99	0.02

S.D., standard deviation; *k*_*a*_, transfer rate from digestive tract (DT) compartment to free water deuterium (FWD) and organically bound deuterium (OBD) compartments (d^−1^); *k*_1_, transfer rate from OBD compartment to FWD compartment (d^−1^); *k*_*e*_, excretion rate from FWD compartment (d^−1^); *T*_1*/*2_ *k*_*m*_, half-life of *k*_*m*_ (d); *R*_*r*_, recovery ratio to the administered deuterium; *d*_1_, distribution factor to FWD compartment; *d*_2_, distribution factor to OBD compartment. The structure of the OBD model and the parameters are shown in Fig. 1. Parameter values were determined by the least squares method.

**Table 4:** Estimated parameters of OBD model for each volunteer administered with D-labelled palmitic acid.

Parameter	Volunteer														
	Male							Female						All	
	1	2	3	4	5	Average	S.D	1	2	3	4	Average	S.D	Average	S.D
*d*_1_	0.49	0.51	0.49	0.43	0.45	0.47	0.03	0.36	0.29	0.43	0.25	0.33	0.08	0.41	0.09
*d*_2_	0.51	0.24	0.23	0.57	0.42	0.40	0.15	0.55	0.59	0.21	0.27	0.41	0.19	0.40	0.16
*k*_*a*_ (d^−1^)	0.93	0.91	2.1	2.0	1.8	1.5	0.6	0.79	12	1.4	1.7	4.0	5.4	2.6	3.6
*k*_*e*_ (d^−1^)	0.059	0.055	0.078	0.063	0.040	0.059	0.014	0.11	0.099	0.059	0.064	0.083	0.025	0.070	0.022
*k*_1_ (d^−1^)	0.0070	0.0077	0.0086	0.0019	0.0017*	0.0063	0.0031	0.010	0.0024	0.013	0.013	0.0097	0.0051	0.0080	0.0042
*T*_1*/*2_* ka* (d^−1^)	0.75	0.76	0.33	0.35	0.39	0.51	0.22	0.88	0.057	0.50	0.40	0.46	0.34	0.49	0.26
*T*_1*/*2_* k*_*e*_ (d^−1^)	12	13	8.9	11	17	12	3	6.3	7.0	12	11	9.0	2.7	11	3
*T*_1*/*2_* k*_1_ (d^−1^)	98	90	80	373	–	161	142	67	294	52	53	117	119	138	125
*Rr*	1.0	0.76	0.72	1.0	0.87	0.87	0.13	0.92	0.89	0.64	0.53	0.74	0.19	0.81	0.16
Dose coefficient (× 10^–11^ Sv Bq^−1^)	10	5.4	4.2	36	22	16	13	7.3	29	3.2	3.5	11	12	13	12

S.D., standard deviation. *k*_*a*_, transfer rate from digestive tract (DT) compartment to free water deuterium (FWD) and organically bound deuterium (OBD) compartments (d^−1^); *k*_1_, transfer rate from OBD compartment to FWD compartment (d^−1^); *k*_*e*_, excretion rate from FWD compartment (d^−1^); *T*_1*/*2_* k*_*m*_, half-life of *k*_*m*_ (d); *R*_*r*_, recovery ratio to the administered deuterium; *d*_1_, distribution factor to FWD compartment; *d*_2_, distribution factor to OBD compartment. The structure of the OBD model and the parameters are shown in Fig. 1. Parameter values were determined by the least squares method except for the value accompanied with asterisk. *Presumed value of data that did not show a clear indication of the second component of exponential decrease.

**Table 5:** Estimated parameters of OBD model for each volunteer administered with D-labelled soybean.

Parameter	Volunteer												
	Male							Female				All	
	1	2	3	4	5	Average	S.D	1	2	Average	S.D	Average	S.D
*d*_1_	0.59	0.61	0.70	0.56	0.62	0.62	0.05	0.41	0.52	0.47	0.08	0.58	0.09
*d*_2_	0.26	0.24	0.29	0.44	0.16	0.28	0.10	0.0	0.28	0.14	0.19	0.24	0.14
*k*_*a*_ (d^−1^)	2.7	4.7	2.8	3.0	2.4	3.1	0.9	2.6	3.8	3.2	0.8	3.1	0.81
*k*_*e*_ (d^−1^)	0.10	0.058	0.041	0.055	0.064	0.063	0.022	0.074	0.063	0.068	0.008	0.065	0.019
*k*_1_ (d^−1^)	0.035	0.011	0.0022	0.0034	0.013	0.013	0.013	–	0.0012	0.0012	–	0.011	0.013
*T*_1*/*2_* ka* (d^−1^)	0.25	0.15	0.25	0.23	0.29	0.23	0.05	0.27	0.18	0.22	0.06	0.23	0.05
*T*_1*/*2_* k*_*e*_ (d^−1^)	6.9	12	17	13	11	12	4	9.4	11	10	1	11	3
*T*_1*/*2_* k*_1_ (d^−1^)	20	61	311	205	53	130	124	–	578	578	–	205	214
*Rr*	0.86	0.85	1.0	1.0	0.78	0.90	0.10	0.41	0.80	0.61	0.28	0.81	0.20
Dose coefficient (× 10^–11^ Sv Bq^−1^)	1.9	4.2	18	17	2.9	8.7	7.8	N.D	N.D	–	–	–	–

S.D., standard deviation. *k*_*a*_, transfer rate from digestive tract (DT) compartment to free water deuterium (FWD) and organically bound deuterium (OBD) compartments (d^−1^); *k*_1_, transfer rate from OBD compartment to FWD compartment (d^−1^); *k*_*e*_, excretion rate from FWD compartment (d^−1^); *T*_1*/*2_* k*_*m*_, half-life of *k*_*m*_ (d); *R*_*r*_, recovery ratio to the administered deuterium; *d*_1_, distribution factor to FWD compartment; *d*_2_, distribution factor to OBD compartment. The structure of the OBD model and the parameters are shown in Fig. 1. Parameter values were determined by the least squares method. N.D., not determined.

**Figure 7:**
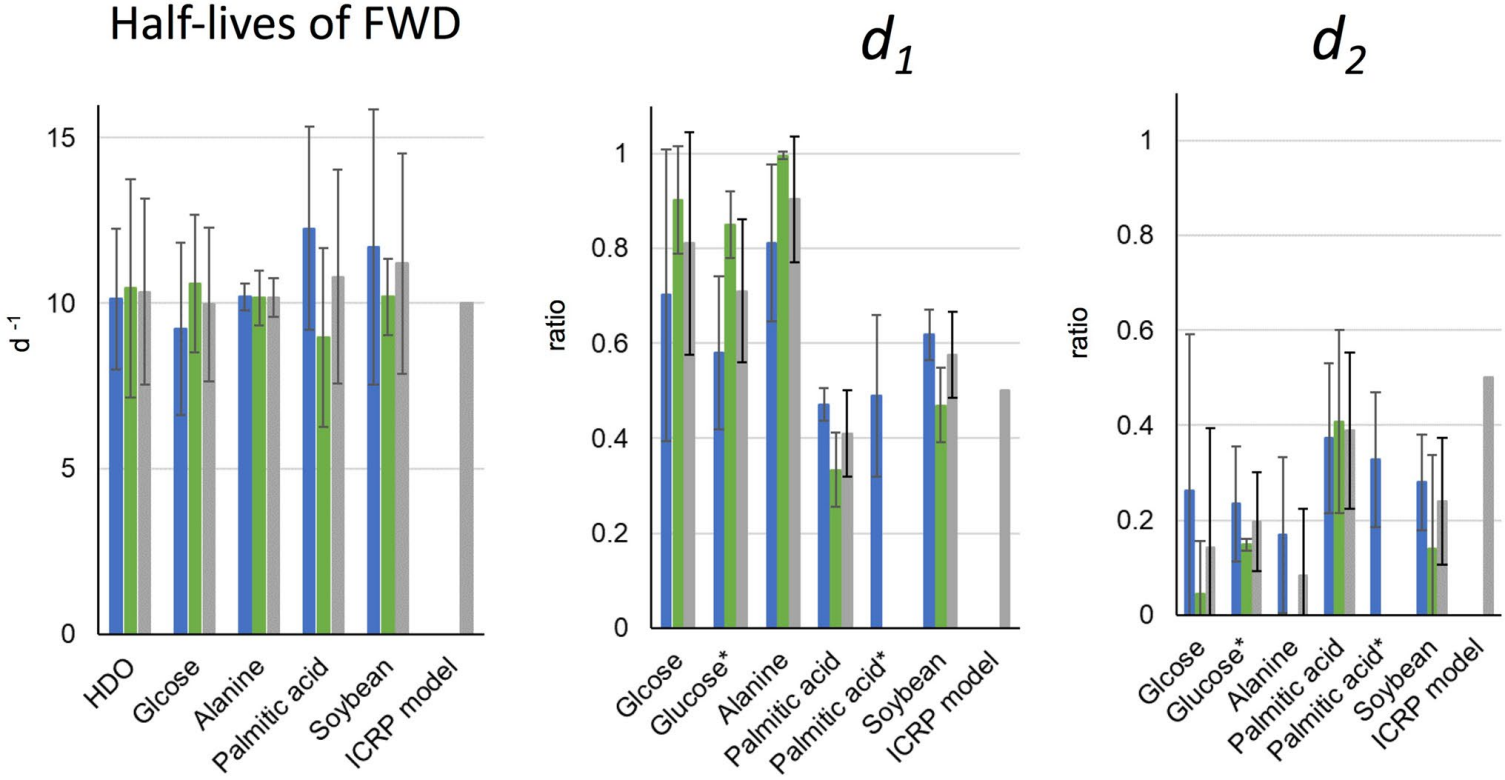
Biokinetic parameters of the OBD metabolic models. The model structure is shown in Fig. 1. Half-lives of FWD, the half-life of deuterium (D) in free water D (FWD) compartment among volunteers of each group; *d*_1_, distribution factor to FWD; *d*_2_, distribution factor to OBD compartment; blue, male volunteers; green, female volunteers; grey, all volunteers. Error bar shows the standard deviation among volunteers in each group. *The values obtained from ^13^C-labelled compound administration experiments^31^. In those case, the ratios of the first and the second component of the exponential decrease in ^13^C/^12^C ratios in breath corresponded to *d*_1_ and *d*_2_, respectively.

The mean half-lives and standard deviations for the free water deuterium (FWD) compartment of the HDO models, as estimated from the data of D_2_O-administered males (n = 3), females (n = 6), and the combined group of volunteers, were 10 ± 2, 10 ± 3, and 10 ± 3 days, respectively (Fig. 7). These values were comparable to the reported half-lives of the FWT described above and to the value given by the ICRP OBT model. These results support the assumption that the biokinetics of OBD are equivalent to that of OBT.

The half-lives of the OBD compartment in the HDO models were estimated using data from 1 of 3 male and 3 of 6 female D_2_O-administered volunteers. The second component of the exponential decrease was not observed in the remaining volunteers in the group. The values obtained varied from 44 to 107 days. Balonov et al. studied the metabolism of HTO after its injection, inhalation, and ingestion, and reported that the second component of HTO decreased with a half-life ranging from 39 to 76 days^29^. Trivedi et al. (1997) reported a longer half-life, ranging from 58 to 104 days, in urine from workers exposed to HTO^30^. Our results are comparable to these, although the longer half-lives of the second component were considered to be affected by the observation period.

### Half-lives of the FWD compartment in the OBD models

The half-lives of the FWD compartment in the OBD models estimated using data from volunteers administered with D-labelled glucose (n = 11), alanine (n = 6), palmitic acid (n = 9), and soybean (n = 7), were 10 ± 2, 10 ± 1, 11 ± 3, and 11 ± 3 days, respectively (Fig. 7). The difference between male and female volunteers was not significant (*p* > 0.05) for either compound or food. The mean half-life of FWD for each compound and food did not vary significantly (*p* > 0.05). Values were approximately comparable to half-lives of the fast-decreasing component, reported from experiments administering HTO^6–13^ and OBT^20^ described above, and the value used in the ICRP OBT model^5^. These results indicate that the half-lives of FWD in the OBD models were independent of the metabolic degradation rates of ingested compounds and foods, and corresponded to the half-life of FWD in each volunteer.

### Distribution ratios (*d*_1_) to the FWD compartment in the OBD models

The mean distribution ratios (*d*_1_) for glucose, to the FWD compartment, determined in males (n = 5), females (n = 6), and in the combined group, were 70 ± 31%, 90 ± 11%, and 81 ± 23%, respectively (Fig. 7). In a previous study, the biokinetics of organic carbon were investigated using ^13^C-labelled compounds, including the administering of ^13^C-labelled glucose to males and females, and ^13^C-labelled palmitic acid to male volunteers^31^. The ratios reported from this previous study were approximately equivalent to ours: male, 58 ± 16%; female, 85 ± 7%; and all, 71 ± 15% (Fig. 7).

The mean distribution ratios (*d*_1_) determined from the data in male (n = 5) and female (n = 4) volunteers in the palmitic acid group were 47 ± 3% and 33 ± 8%, respectively. The ratio for male was comparable to the ratio found for male volunteers treated with ^13^C-labelled palmitic acid (49 ± 17%) in the previous paper (Fig. 7). These results exhibit the same metabolic rate for each compound, independent of the isotope label used. The difference in the distribution ratios between male and female volunteers administered palmitic acid was significant (*p* < 0.01). The ratio of body fat to body weight in a healthy Japanese aging from 20 to 29 years is larger in females (28.4 ± 7.0%) than that in males (18.7 ± 6.6%)^32^. The cause of the smaller *d*_1_ value in female volunteers was presumed to be the larger transfer ratio to body fat due to the larger mass of body fat compared with that for male volunteers. Melintescu et al. have estimated significantly larger retention of tritium from OBT intake for females than for males due to the higher adipose mass in their physiologically based multicompartment model^33^. Our results were consistent with their estimations.

The mean distribution ratios (*d*_1_) to the FWD compartment, estimated from the data in D-labelled alanine-administered male (n = 3) and female (n = 3) volunteers were 81 ± 14% and 100 ± 0%, respectively. The metabolism differs between amino acids. From previous experiments involving the administration of ^13^C-labelled compounds, the mean distribution ratios of amino acids were obtained from male and female volunteers as follows: glutamic acid, 64 ± 9% and 63 ± 45%, respectively; glycine, 36 ± 4% and 40 ± 1%, respectively; phenylalanine, 31 ± 3% and 40 ± 4%, respectively; and leucine, 45 ± 6%, obtained from male volunteers only^31^. Our data for alanine gave much larger ratios than these amino acids (*p* < 0.01).

Using the *d*_1_ of glucose, alanine, and palmitic acid described above, the *d*_1_ of soybean was estimated to be a weighted mean of these ratios according to the nutritional balance of green soybean, in the assumption that these compounds represent carbohydrates, proteins, and lipids, respectively. The estimated distribution ratio (*d*_1_) for soybean based on these compounds (73 ± 8%) was greater than that obtained by the D-labelled soybean administration experiment (58 ± 9%). The reason for the difference was presumed to be the representativeness of alanine and palmitic acid as amino acid and lipid. The ratio of alanine was greater than the ratios of other amino acids, as described above, and the ratio of palmitic acid was greater than the reported ratio of unsaturated fatty acids obtained by the ^13^C-labelled experiment in a previous paper as follows: oleic acid, 35 ± 11%; linoleic acid, 29 ± 12%^31^. Our estimation of ratio (53 ± 7%) improved when we additionally considered the ratios obtained by the ^13^C-labelled compound administration experiments (see “Methods”).

The procedure used to estimate the distribution ratio of soybean, which utilised the ratios of various compounds estimated by both D- and ^13^C-labelled compound administration experiments, was applied to the estimation of dietary reference intakes, to validate the ratio in the ICRP model. The ratios calculated for reference intakes in Japan^34^ and the United States^35^, with a relatively high carbohydrate and high fat content, respectively, were 68 ± 15% and 66 ± 14%. These values were slightly higher than those in the ICRP model were (50%). The estimated dose coefficients, from the ICRP model, when the ratio of 50% is replaced by the ratios of 68% and 66%, were 3.3 × 10^–11^ and 3.4 × 10^–11^ Sv Bq^−1^, respectively. These values are slightly smaller than the dose coefficient used in the ICRP model (4.2 × 10^–11^ Sv Bq^−1^), and suggest that the ratio of 50% adopted by the ICRP was cautious.

The distribution ratio in the D-labelled soybean administration experiment (48 ± 16%) was approximately comparable to the distribution ratio to the FWT compartment in the ICRP model. In rats, the cumulative excretion of tritium in urine, during continuous feeding on tritiated rice, was greater than that shown in tritiated soybean^15^. Obtaining such data in humans for other major foods may help to support the ratio used in the ICRP model.

### Half-lives of the OBD compartment in OBD models

The second component of the exponential decrease was not observed in some volunteers in the glucose and alanine groups (Figs. 2, 3). The distribution ratios *d*_1_ in the OBD models of glucose and alanine were 81 ± 23% and 90 ± 13%, respectively. In those volunteers, the second component seemed to be below the background fluctuation, since most of the administered OBD was considered to be immediately degraded to FWD. Further studies are necessary to clarify the half-lives of OBT, for such molecules.

The second component was observed in almost all volunteers in the palmitic acid and soybean groups, except for one female volunteer in the soybean group. The half-life of the OBD compartment was calculated from *k*_1_ in each volunteer. The half-lives varied markedly among volunteers, as follows: males treated with palmitic acid, 80–405 days; females treated with palmitic acid, 52–294 days; and males treated with soybean, 20–311 days. Since (in all but one case in each group) the ratios were above the background fluctuation throughout the experimental period, the reason for the difference seemed to be due to the higher variability among individuals in the half-life of OBT, instead of errors derived from experimental detection limits. Longer half-lives, of several hundred days, have been reported in studies on urine from clock dial painters exposed to tritiated luminous compounds^36^. Taylor, in 2003, proposed a dosimetry model involving a third component with a half-life of 350 days^37^. The ICRP also adopted a second OBT compartment, with a longer half-life of 365 days, in its model for workers^4^. This work first provided the half-lives of the second component, estimated for OBT in major nutritional molecules and foods, including half-lives of several hundred days in some volunteers. These results suggest the necessity for a long half-life compartment in dosimetry models for OBT.

### Estimation of committed effective doses for palmitic acid and soybean

The committed effective doses from 1 Bq of tritium were estimated by the OBD models of males and females in the palmitic acid group and males in the soybean group. The values varied from 4.2 × 10^–11^ to 3.5 × 10^–10^ Sv Bq^−1^, 3.2 × 10^–11^–2.9 × 10^–10^ Sv Bq^−1^ and 1.9 × 10^–11^–1.8 × 10^–10^ Sv Bq^−1^, among respective groups. The recovery ratios of *Rr* (*d*_1_ + *d*_2_) in the palmitic acid and soybean groups were 0.81 ± 0.16 and 0.90 ± 0.10, respectively. Since the committed effective doses from the unrecovered fraction were not included in the estimates, the true values would be higher. The values for palmitic acid and soybean were higher than the dose coefficient used by the ICRP, for members of the public (4.2 × 10^–11^ Sv Bq^−1^). In rats, cumulative residual amounts of OBT, after the administration of tritiated fatty acids, were higher than those from glucose were^17^, and that from soybean was higher than that from rice and wheat, which mainly consisted of carbohydrate^15^. In humans, the estimated committed effective dose from 1 Bq of ^14^C in fatty acids was greater than that from glucose^31^. This work provides the first estimates of the committed effective dose from OBT in major nutritional molecules and food. They were, however, considered compound and ingredient specific. In our study, since the committed effective doses for glucose and alanine were not obtained, due to the lack of reliable *d*_2_ and *k*_1_ in the OBD models, we could not find the effective dose for a reference diet by the weighted mean of each nutrient molecule. Therefore, further study is necessary to validate the recommended dose coefficient of the ICRP, by obtaining comprehensive data regarding the metabolism of OBT for various nutrient molecules and foods, such as proteins, carbohydrates, and grains.

## Conclusions

For reference diets in Japan and the United States, the estimated distribution ratios of ingested OBT to be immediately metabolised to FWT were slightly greater than the value assumed in the ICRP OBT model, which has been adopted for the calculation of dose coefficient. These results suggest that the assumed value, used in the ICRP model, was cautious from the viewpoint of radiation safety. However, the half-life of the OBT compartment, in each volunteer administered palmitic acid and soybean, varied from dozens to several hundred days. These results provide the estimated half-lives of OBT for major nutritional molecules and food. For dose estimation, the need to consider long half-lives of over 100 days was suggested. Consequently, the committed effective doses for 1 Bq of tritiated palmitic acid and soybean were greater than the dose coefficient value used for members of the public, recommended by the ICRP (4.2 × 10^–11^ Sv Bq^−1^). These results suggest that OBT, for some ingredients, might give higher doses than the current dose coefficient suggests. However, we were unable to obtain the dose for the reference diets. Therefore, to validate the dose coefficient by the ICRP, further studies are necessary to estimate the committed effective doses from other major nutrient molecules and foods. In addition to that, further studies on various age groups are deemed necessary, because our findings are based on data from volunteers aging from 20 to 28.

## Methods

### Ethical considerations

All experimental protocols in the current study conform to the ethical guidelines of the 1975 Declaration of Helsinki^38^ and were approved by the Review Board for Human Subject Experiments established by the Institute for Environmental Sciences, Aomori, Japan. Written informed consent was obtained from each volunteer prior to participation.

### Procedures for the administration of labelled materials

D_2_O, and D-labelled glucose, alanine, palmitic acid, and soybean were administered orally to healthy adult Japanese male and female volunteers. The total number of volunteers was 42, although some dropped out of the experiment, for various reasons. The data from these volunteers were eliminated. This study lasted 4 years; administration experiments were conducted from September to January, and each volunteer participated in one of the 4 years. The numbers of volunteers in each group, their ages, and body weights are shown in Table 6.

**Table 6:** Volunteer groups.

Volunteer group	Administration	Sex	Age (years)		Body weight (kg)		N
			Mean	S.D	Mean	S.D	
D_2_O	D_2_O	M	22.3	2.5	83.7	14.4	3
		F	21.5	3.2	52.0	6.1	6
Glucose	D-labelled glucose	M	21.4	0.5	66.1	6.3	5
		F	23.4	3.1	53.8	4.7	6
Alanine	D-labelled alanine	M	20.7	1.2	62.6	4.8	3
		F	21.3	1.2	53.0	4.0	3
Palmitic acid	D-labelled palmitic acid	M	20.8	0.5	63.6	13.2	5
		F	20.3	0.5	47.1	5.1	4
Soybean	D-labelled soybean	M	20.8	0.4	62.6	4.7	5
		F	21.5	0.7	52.7	0.3	2

M, male. F, female. S.D., standard deviation.

D_2_O and D-labelled glucose solutions were administered once orally, at 12:15, immediately before eating lunch on experimental day 0. D-labelled alanine and palmitic acid were mixed with food and administered once daily for four successive days, at 12:15. The administration period for D-labelled soybean lasted 12 days, with two series of 5 consecutive days of administration, in the same manner, with an interval of 2 days. For each volunteer administered D-labelled compounds, the same compound labelled with ^13^C was given simultaneously in each administration, apart from three male and three female volunteers who were not administered ^13^C-labelled glucose alongside their D-labelled glucose.

### Preparation of labelled compounds and soybean

All D-labelled and ^13^C-labelled compounds were purchased from Cambridge Isotopes, Inc. (Tewksbury). In each D-labelled molecule, hydrogen bound to carbon was uniformly replaced by D. Such D is not exchangeable to hydrogen in free body water after administration until it is metabolised to HDO, and is called non-exchangeable OBD (Nx-OBD). Exchangeable hydrogen, which was not bound to carbon, such as hydroxyl group hydrogen, was not replaced by D. In each ^13^C-labelled compound, ^13^C was uniformly labelled.

D-labelled soybean was obtained by hydroponic culture with 20% D/H water. The D-labelled soybean was harvested three times. The harvested soybeans were boiled, freeze-dried, and then homogenised. The soy powder product was orally administered. To measure the D/H ratio, the soy powder was first immersed in drinking water, to replace exchangeable OBD (Ex-OBD), which was not bound to carbon. After the second freeze-drying process, the D/H ratio was measured using a mass spectrometer (Iso Prime 100; Elementar, Langenselbold, Germany). Buried tritium is defined as the tritium in a large biomolecule that remains in the molecule after rinsing samples with tritium-free water, despite its exchangeable position in the molecule^39^. A fraction of buried deuterium was considered to remain in soybean powder. However, the ratio of buried tritium has been estimated as less than 5% compared to the non-exchangeable OBT present in washed plant samples^40^. Therefore, we considered that effect of the amount of buried deuterium on the measured D/H ratio of soy powder was negligible. The hydrogen content of the soybean powders (gH/gDW) was measured using a gas chromatograph (NCH-22F; Sumika Chemical Analysis Service, Oosaka, Japan). The OBD content of the soybean powders (gOBD/gDW) were calculated from their D/H ratio and hydrogen content, and are summarised in Table 7.

**Table 7:** Characterization of D-labelled soybean.

Harvest number	Harvested crop weight	H content	D/H ratio	D content
	gDW	gH/100 gDW	%	gOBD/100 gDW
1	189.0	7.2 ± 0.2	12.0 ± 0.5	1.7 ± 0.1
2	222.8	7.1 ± 0.7	10.1 ± 0.8	1.4 ± 0.2
3	313.3	7.4 ± 0.2	9.8 ± 0.5	1.5 ± 0.1

H, hydrogen. D, deuterium. DW, dry weight. OBD, organically bound deuterium. Values are presented as mean ± standard deviation.

### Dosage of D and ^13^C

Dosages of D and ^13^C in labelled compounds and foods are summarised in Table 8. The doses of D for the volunteer groups administered HDO and D-labelled glucose were 1.1 and 0.51 g per person, respectively. The total dosage of D for the groups of D-labelled alanine and palmitic acid were 0.34 and 0.85 g per person, respectively, over four successive days of administration. The D-labelled soybean administration experiments were conducted three times, corresponding to three harvests, and the resulting dosages of D in these experiments were 1.6 (n = 2), 1.6 (n = 3), and 1.2 (n = 3) g per person, over four days.

**Table 8:** Amounts of administered materials.

Volunteer group	D-labelled compound or food			^13^C-labelled compound		
	Administration	g person^−1^	gNx-OBD person^−1^	Administration	g person^−1^	g^13^C person^−1^
D_2_O	D_2_O	5.5	1.1	–	–	–
Glucose	D-labelled glucose	7.0	0.51	^13^C-labelled glucose	3.0	1.2
Alanine	D-labelled alanine	4.0	0.34	^13^C-labelled alanine	0.40	0.17
Palmitic acid	D-labelled palmitic acid	4.0	0.85	^13^C-labelled palmitic acid	0.40	0.30
Soybean	D-labelled soybean harvest 1	90	1.6	–	–	–
	D-labelled soybean harvest 2	110	1.6	–	–	–
	D-labelled soybean harvest 3	85	1.2	–	–	–

*Nx-OBD* non-exchangeable organically bound deuterium.

### Sample collection and determination of D/H and ^13^C/^12^C ratio

Urine and breath samples were periodically collected from days − 7 to 119, where the first day of the administration was defined as day 0. Urine samples were collected daily, when the volunteers rose in the morning, until day 28, then every two or three days until day 119. Additional samples were collected frequently, after administration. Urine samples were collected at every urination until bedtime, on days of administration. Breath samples were collected immediately prior to the administration, every 20 min from 12:30 to 17:10, and then hourly from 18:00 until bedtime.

Urine was distilled, and the condensed water was collected for the measurement of D/H ratio, using a mass spectrometer (Iso Prime 100; Elementar, Langenselbold, Germany). Breath samples were collected using an airtight bag. The samples were analysed within 3 days. After purifying the CO_2_ in the breath using a gas chromatograph (MAT GC; Thermo Electron, Waltham, MA, USA), the ^13^C/^12^C ratio was measured using a mass spectrometer (Dalta V Advantage; Thermo Electron, Waltham, MA, USA).

The increments were calculated of D/H and ^13^C/^12^C ratios above the background values measured for the samples before administration. The ratios were normalised to the same dosage (1 g D or ^13^C per 70 kg body weight). When the D/H or ^13^C/^12^C ratios were below the limits (90% upper confidence limits of the background fluctuation), to cut off the data and any subsequent data were discarded.

### Development of the models

A metabolic model was developed, for each volunteer. For volunteers in the glucose, alanine, palmitic acid, and soybean groups, the model structure of the OBD shown in Fig. 1 was adopted, while for volunteers in the D_2_O group, the model structure for HDO was adopted. For each model, parameters were determined using the least squares method, using Microsoft Excel version 2007. The detailed procedures have been described previously^31^.

Data below the detection limit were discarded, and the second component was not clearly discernible in 3 of 42 volunteers, e.g., the data from the female subject in the palmitic acid group are shown in Fig. 3, in red. In this case, *k*_1_ was not determined and other parameters were determined under the condition that *k*_1_ was fixed at 0.0017 d^−1^. The value was derived from the mean residence time of carbon (1.6 years) in adipose tissue, having one of the slowest metabolic rates among human organs^41^. The value was considered the same as that of OBD in adipose tissue.

### Estimations of the parameter values for food and reference diet by the values for various compounds

The *d*_1_ for soybeans was estimated by the weighted mean of the ratios obtained from administration experiments with D-labelled compounds, or with D-and ^13^C-labelled compounds, corresponding to their known nutritional composition in boiled soybean^42^, assuming the nutritional balance of boiled soybean remained the same before and after the immersion process. The combinations of the examined molecules, which were the representative molecules and their assumed corresponding nutrition, are shown in Table 9. DNA precursors were not taken into consideration. To validate the distribution ratio (50%) to the HTO compartment, in the ICRP model, ratios for reference diets were calculated in the same manner as nutritional surveys conducted in Japan^43^ and the United States^35^, as examples of relatively high carbohydrate and high fat diets, respectively. The composition of amino acids in protein in the reference diet was adopted from Iwaya [44].

**Table 9:** Representative molecules for each nutrient.

Representative molecule	Corresponding nutrition	*d*_1_*	Experiment**
Glucose	Carbohydrate	0.81 ± 0.23	D-labelled glucose
Alanine	Alanine	0.90 ± 0.13	D-labelled alanine
Glycine	Glycine	0.29 ± 0.04	^13^C-labelled glycine^31^
Leucine	Branched chain amino acids	0.33 ± 0.03	^13^C-labelled leucine^31^
Phenylalanine	Aromatic amino acids	0.25 ± 0.04	^13^C-labelled phenylalanine^31^
Glutamic acid	Other amino acids	0.61 ± 0.06	^13^C-labelled glutamic acid^31^
Palmitic acid	Saturated fatty acids	0.41 ± 0.09	D-labelled palmitic acid
Oleic acid	Mono-unsaturated fatty acids	0.35 ± 0.11	^13^C-labelled oleic acid^31^
Linoleic acid	Poly-unsaturated fatty acids and others	0.29 ± 0.12	^13^C-labelled linoleic acid^31^

*Ratio of organic compounds to inorganic compounds immediately after administration.

**Isotope administration experiments to determine the value of *d*_1_. Values of *d*_1_ are mean ± standard deviation.

### Calculation of dose coefficient

The 50-year cumulative burden of tritium, after ingestion, was estimated for each volunteer, using the OBD model developed in this study, under the assumption that tritium has the same metabolic behaviour as deuterium. The committed effective dose from ingested OBT for each volunteer was calculated from the 50-year cumulative burden, according to the procedure published by the ICRP, using a tissue-weighting factor of unity, under the assumption of uniform distribution of tritium throughout the body^5^. Although the ratio of recovery to administration was not unity in some volunteers, the unrecovered fraction was not considered in the estimation of cumulative burden. Therefore, the estimated committed effective dose would increase when the unrecovered fraction is included.

## Data Availability

The datasets generated and analysed during the current study are available from the corresponding author, on reasonable request.
